# Design and implementation of Cs/GO/TiO_2_ nanocomposite for controlling sulfamethoxazole

**DOI:** 10.1038/s41598-026-44482-9

**Published:** 2026-04-08

**Authors:** Khaled S. Amin, Mahmoud S. Ghanem, Mohamed M. Mahmoud, Amged G. El-Srougy, Hanan Elhaes, Medhat A. Ibrahim

**Affiliations:** 1 Physics Department, Faculty of Science, Al-Azhar University, Cairo, Egypt; 2 Physics Department, Faculty of Science, Mansoura University, Mansoura, Egypt; 3 Physics Department, Faculty of Women for Arts, Science and Education, Ain Shams University, 11757 Cairo, Egypt; 4 Spectroscopy Department, National Research Centre, 33 El-Bohouth St., 12622, Dokki, Giza, Egypt; 5 Center for Converging Sciences and Emerging Technologies (CoSET), Benha National University (BNU), El-Obour, 13518 Egypt; 6 Molecular Modeling and Spectroscopy Laboratory, Centre of Excellence for Advanced Science, National Research Centre, 33 El-Bohouth St., 12622, Dokki, Giza, Egypt

**Keywords:** DFT, Graphene oxide (GO), Titanium dioxide (TiO_2_), Sulfamethoxazole, Total density of states, QTAIM, Chemistry, Environmental sciences, Materials science, Nanoscience and technology

## Abstract

The persistent presence of sulfamethoxazole (SMX), a widely used antibiotic, in aquatic environments poses significant ecological and health risks. This issue stems largely from its incomplete removal by conventional wastewater treatment processes. In this study, density functional theory (DFT) calculations were employed to investigate the adsorption behavior of hydrated SMX on chitosan/graphene oxide/titanium dioxide (Cs/GO/TiO_2_) composites. Two adsorption configurations were examined: interaction via the amine (–NH_2_) group of chitosan and coordination between the isoxazole nitrogen of SMX and TiO_2_. Electronic and topological properties were analyzed using total dipole moment (TDM), HOMO–LUMO energy gap (ΔE), molecular electrostatic potential (MESP), global reactivity descriptors (GRDs), density of states (TDOS/PDOS/OPDOS), quantum theory of atoms in molecules (QTAIM), and non-covalent interaction (NCI) analyses. The results reveal increased polarity, reduced energy gaps, and notable charge redistribution upon adsorption. The calculated adsorption energies (–2.31 eV and − 3.69 eV) indicate energetically favorable interactions, with relative stability depending on the adsorption site. These findings provide atomic-level insight into SMX–composite interactions and highlight the potential role of Cs/GO/TiO₂ composites in adsorption-based removal of antibiotic contaminants.

## Introduction

Pharmaceutically active compounds pose a significant challenge, as conventional wastewater treatment systems continuously release them into aquatic ecosystems, endangering both human health and the environment^1^. Antibiotic use has increased over the past 20 years, but there are still few guidelines regarding the amount used or the disposal of leftover antibiotics^2^. Sulfamethoxazole (SMX), a type of these antibiotics used for the prevention and treatment of bacterial infections in the urethra and other tissues, is commonly detected in aquatic environments due to wastewater treatment plants, improper drug disposal, and waste streams from antibiotic production, posing compounded ecological risks due to its synergistic toxicity and results of its persistence and incomplete removal by conventional treatment methods^3–5^.

In order to eliminate the contamination caused by SMX, a number of advanced materials have been explored as adsorbents and photocatalysts, including Chitosan (Cs), graphene oxide (GO) and composites based on titanium dioxide (TiO_2_)^6–8^. Cs is a biodegradable, low-cost polymer with promising adsorption properties for antibiotic removal^9,10^. Deacetylation of chitin yields the natural polymer chitosan, a derivative of chitin. Cs, the second most prevalent polysaccharide in nature after cellulose, is distinguished by its degradability, non-toxicity, biocompatibility, and biological safety^11^. The amino groups of the polymer chains are protonated under acidic conditions, which gives the polymer a cationic nature. The given peculiarity enables Cs to interact with a wide range of molecules, thus becoming the only cationic marine polysaccharide^12^. GO has emerged as an auspicious material for removing pharmaceutical contaminants, including the antibiotic SMX, from water systems due to its unique physicochemical properties, such as its large surface area, abundance of functional groups containing oxygen, and high dispersibility in water, making it an effective adsorbent for a variety of pollutants^8,13^. Combinations with GO increase adsorption capacity, due to synergistic surface interactions^14^, ^15^. Functionalizing GO with Cs not only improves the mechanical strength of the composite but also increases its adsorption efficiency^10^. TiO_2_ is a wide-bandgap semiconductor with structural stability and favorable electronic properties and exists in three phases: Anatase, Rutile, and Brookite, with band gaps typically reported as: 3.2, 2.96, 3.02 eV, respectively^16,17^. Photocatalytic degradation using TiO_2_ is a promising, eco-friendly, and highly efficient method for the control of recalcitrant organic pollutants, especially of sulfonamide antibiotics (such as SMX), which is advantageous, especially in places where bio/physical remediation techniques are not efficient enough^18^. In brief, adding TiO_2_ to Cs and GO composites provides a synergistic effect, combining photocatalytic degradation (from TiO_2_) with enhanced adsorption (from Cs and GO), leading to improved removal efficiency, selectivity, and practical handling for SMX in water treatment applications^15,19,20^. These composite systems offer a promising way of degrading the recalcitrant contaminants, used as an example, NF_2_, using sulfamethoxazole (SMX), due to the exploitation of these unique physicochemicalties of such composite materials, thus overcoming the limitations inherent to the single-component materials^21–23^.

SMX and other sulfonamide antibiotics can be effectively removed from water using a variety of composite materials, including biochar-based TiO_2_, ZnO-based photocatalysts, and Z-scheme heterojunctions. The majority of research focuses on experimental removal rates and general mechanistic insights, including the role of reactive oxygen species, π-π interactions, and charge transport^24,25^. Although some studies have identified intermediates and proposed degradation pathways for sulfonamide pollutants, little is known about the precise adsorption and degradation mechanisms, particularly at the atomic scale. This discrepancy is especially noticeable for composite systems, where component synergistic effects (such as charge separation and surface functional groups) are thought to improve removal efficiency but are not fully understood theoretically^24–26^.

Understanding the fundamental interactions at the atomic level is essential for designing and optimizing advanced composite materials for pollutant removal. When SMX is adsorbed and degraded on Cs/GO/TiO_2_ composites, density functional theory (DFT) calculations can provide valuable insights into adsorption energies, preferred binding sites, and electronic structure modifications. Those theoretical observations are very crucial in authentication of empirical evidence and control the systematic creation of more effective functional materials^24–26^.

Several previous investigations have successfully integrated these three components into a single multifunctional composite to exploit their complementary adsorption and photocatalytic properties. The composite systems of Cs/GO/TiO_2_ have been indicated to have high structural stability, ability to separate charges and also high ability to remove organic dye and pharmaceutical contaminants in aqueous medium^27–29^. The synergistic interaction among Cs, GO, and TiO_2_ enhances both adsorption and photocatalytic degradation efficiency, making the composite a promising candidate for antibiotic removal applications. In addition, our theoretical and experimental study on Cs/GO/TiO_2_ as gas sensor has indicated strong changes on both electronic and dipolar properties of composite and adsorption onto gas, hence supporting high sensitivity and charge transfer^12^.

Molecular modeling is considered as a powerful computational technique for elucidating the electronic and structural behavior of many complex systems. In particular, Density Functional Theory DFT offered accurate prediction of electronic transitions, charge transfer, and molecular interactions at the atomic scale, complementing experimental studies across various applications. It has been widely used to elucidate adsorption and sensing mechanisms in chitosan/graphene composites for heavy metal removal^30^, graphene quantum dots substituted with group III and V elements^31^, and PLA/GO/NiO electrodes for ammonia sensing^32^. These studies confirm the reliability of DFT in describing interfacial phenomena, chemical reactivity, and adsorption energetics in nanocomposite systems.

Therefore, the current study is conducted to investigate the adsorption mechanism of sulfamethoxazole on the Cs/GO/TiO_2_ composite using DFT. The analysis involves a comprehensive set of electronic and physical parameters, including the total dipole moment (TDM), ΔE, MESP, GRDs, removal energy, total density of states (TDOS), partial density of states (PDOS), overlap PDOS (OPDOS), QTAIM, and NCI analyses. These tools provide a detailed understanding of charge transfer, adsorption stability, and electronic structure evolution during the interaction of SMX with the composite. The findings offer valuable insights for designing efficient Cs/GO/TiO_2_-based adsorbent systems to remove SMX pharmaceutical contaminants from water.

## Calculation details

All quantum chemical calculations were performed using the Gaussian 09 (G09) program package^33^. The molecular structures were optimized on a personal computer using the B3LYP functional, which combines Becke’s three-parameter exchange functional with the Lee–Yang–Parr correlation functional^34–36^, together with the 3-21G basis set. To ensure the reliability of the 3-21G results, single-point energy calculations were carried out at a mixed basis set level, using B3LYP/6-31G(d) for all nonmetal atoms and LANL2DZ for titanium. This approach provided a more accurate estimation of the total energy while maintaining computational efficiency.

Within the same computational framework, TDM, highest occupied molecular orbital (HOMO), and lowest unoccupied molecular orbital (LUMO) energies were evaluated. ΔE, obtained as the difference between HOMO and LUMO energies, along with TDM, was used to interpret the electronic reactivity and interaction tendencies of the studied systems. MESP was generated to visualize reactive sites, while Mulliken population analysis was used for charge distribution. The total density of states (DOS) was plotted to analyze electronic distribution and orbital contributions. GRDs, such as Ionization Potential (IP), Electron Affinity (EA), Chemical Potential (µ), Electronegativity ($\chi$) Chemical Hardness (η), Absolute Softness (S), and Electrophilicity Index (ω)^12^, were calculated using the following equations:

1 $$IP=-{E}_{HOMO}$$

2 $$EA=-{E}_{LUMO}$$

3 $$\chi=-\left(\frac{{E}_{HOMO}+{E}_{LUMO}}{2}\right)$$

4 $$\mu=-\chi$$

5 $$\eta=\frac{{E}_{LUMO}-{E}_{HOMO}}{2}$$

6 $$S=\frac{1}{2\eta}$$

7 $${\upomega}=\frac{{\mu}^{2}}{2\eta}$$

The adsorption (removal) energy of the SMX molecule on the Cs/GO/TiO_2_ composite surface was evaluated using Eq. (8)^37^:

8 $${E}_{\mathrm{ads}}={E}_{\mathrm{system}}-({E}_{\mathrm{adsorbent}}+{E}_{\mathrm{adsorbate}})$$

Solvent effects were not included in this DFT study, where the calculated adsorption energies are used for comparison qualitatively and are model-dependent, which reflect interactions within a cluster representation, rather than a periodic surface. Finally, QTAIM analysis was performed using the Multiwfn program in combination with Visual Molecular Dynamics (VMD) software^38,39^, to characterize the bonding interactions between SMX and the nanocomposite and to gain insight into the electronic stability and bonding nature of the formed complexes. In addition, NCI and Reduced Density Gradient (RDG) analyses were carried out to visualize and compare the weak interaction regions within the pristine composite and after adsorption of SMX, providing a detailed picture of hydrogen bonding, π-interactions, and van der Waals contacts at the interface. For QTAIM, NCI, and RDG analyses, additional single-point calculations were performed using Grimme-type D3 dispersion in Gaussian 09, whereas all geometry optimizations and adsorption energy calculations were performed without explicit dispersion correction. These dispersion-corrected densities were used only for the topological and non-covalent interaction analyses.

## Results and discussion

### Structural modeling and electronic properties

**Fig. 1:**
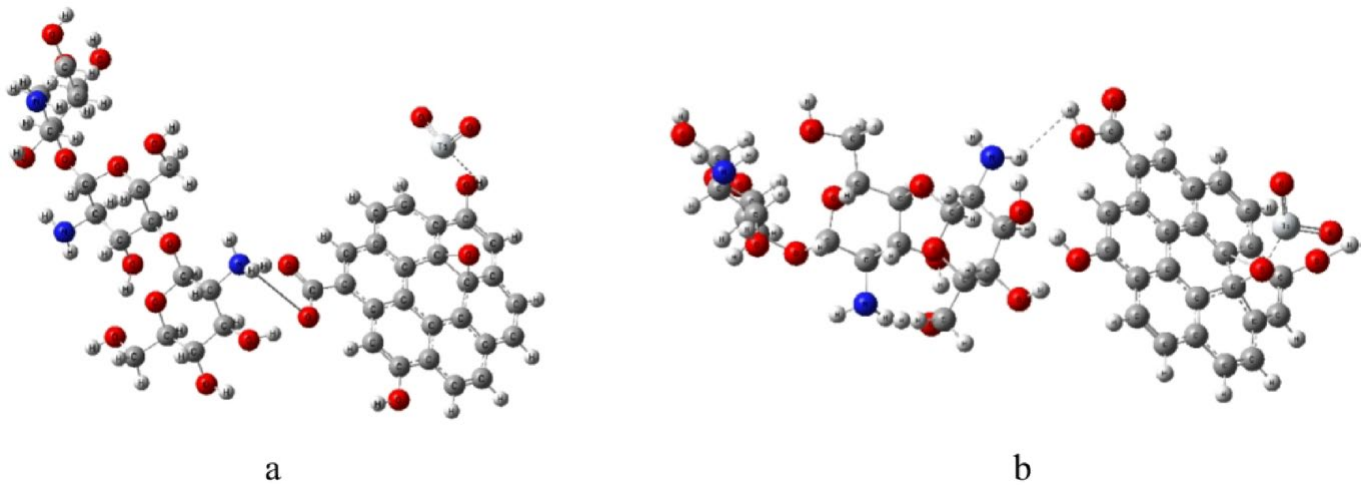
Optimized structures of Cs/GO/TiO_2_ composites: **(a)** interaction through the hydroxyl (–OH) group of GO; **(b)** interaction through the epoxy oxygen (O) of GO. The structures were visualized using GaussView 6.0 (Roy Dennington, Todd A. Keith, and John M. Millam, Semichem Inc., 2016; https://gaussian.com/gaussview6/).

Before performing the DFT calculations, molecular models of the studied composites were constructed. The optimized structures are presented in Fig. 1. The molecular structures were constructed and visualized using the GaussView 6 program^40^. The chitosan (Cs) and graphene oxide (GO) components were modeled based on their typical functional groups, where GO contains carboxyl (–COOH), hydroxyl (–OH), and epoxy (C–O–C) moieties. The Cs/GO composite interacts with TiO_2_ through two possible configurations. In the first configuration (Fig. 1a), TiO_2_ interacts with the hydroxyl (–OH) group of GO, while in the second configuration (Fig. 1b), the interaction occurs through the epoxy oxygen atom of GO.

The total dipole moment (TDM) reflects the overall molecular polarity, which is closely related to charge distribution and intermolecular interactions. Higher TDM values generally indicate enhanced polarity and stronger electrostatic interactions, which can improve adsorption capacity. The HOMO–LUMO energy gap (ΔE) represents the difference in energy between the highest occupied molecular orbital (HOMO) and the lowest unoccupied molecular orbital (LUMO). This parameter is a key indicator of chemical reactivity and electronic transitions. A smaller ΔE corresponds to greater electronic delocalization and higher chemical reactivity, facilitating charge transfer processes^41^.

The structures were optimized at the B3LYP/3-21G level of theory. The calculated TDM and ΔE for both configurations are summarized in Table 1. The Cs/GO/TiO_2_ composite interacting via the –OH group exhibits a slightly smaller energy gap and a higher TDM compared to the epoxy-linked configuration. This suggests that the –OH interaction enhances charge transfer and molecular polarization, leading to improved electronic communication within the composite. This aligns with the enhanced sensitivity previously observed for the same composite when applied as a gas sensor^12^.

**Table 1:** Total dipole moment (TDM) and HOMO–LUMO energy gap (ΔE) for Cs/GO/TiO_2_ composites at the B3LYP/3-21G level.

Structures	TDM (Debye)	ΔE (eV)
**Cs/GO/TiO**_**2**_ **via (OH)**	25.715	0.269
**Cs/GO/TiO**_**2**_ **via epoxy** **(O)**	24.122	0.426

### Interaction mechanism between Cs/GO/TiO_2_ composite and SMX

**Fig. 2:**
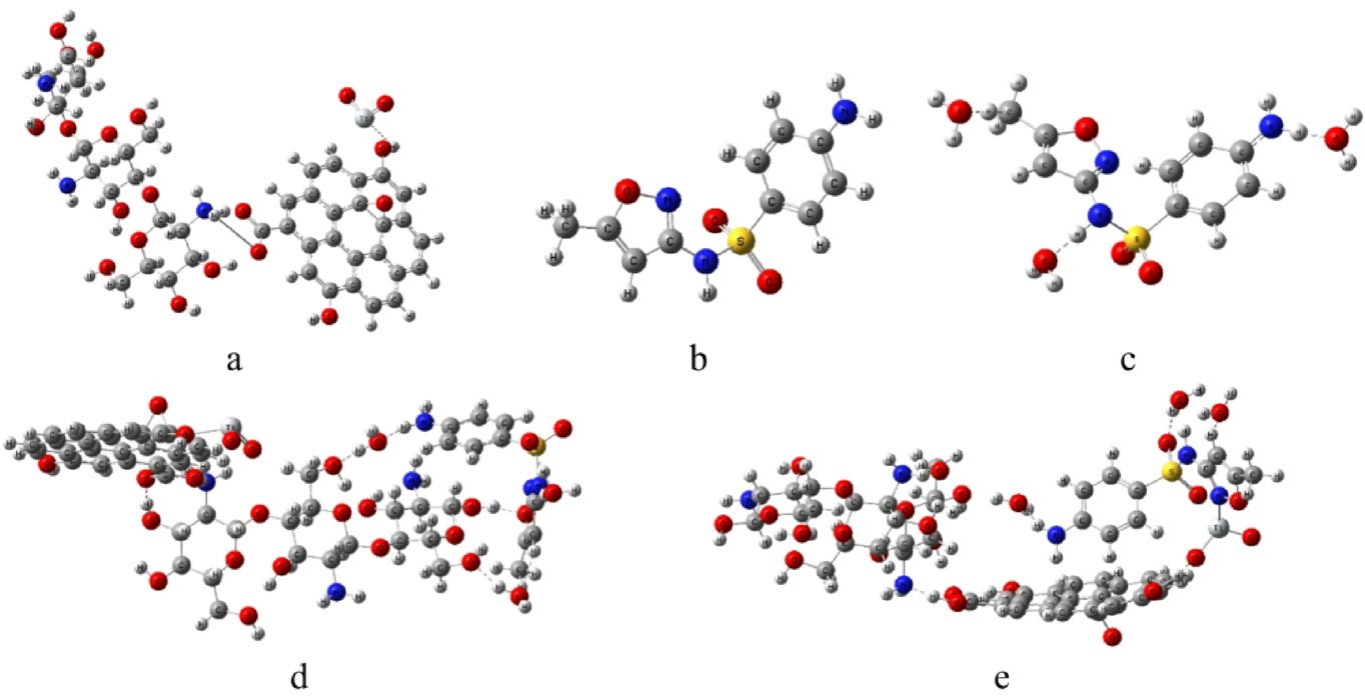
Optimized structures of **(a)** Cs/GO/TiO_2_ composite, **(b)** sulfamethoxazole (SMX), **(c)** Hydrated SMX, **(d)** Cs/GO/TiO_2_ interacting with SMX via the –NH_2_ group of chitosan, and **(e)** Cs/GO/TiO_2_ interacting with SMX via TiO_2_ (N–N-isoxazole interaction). The structures were visualized using GaussView 6.0 (Roy Dennington, Todd A. Keith, and John M. Millam, Semichem Inc., 2016; https://gaussian.com/gaussview6/).

To validate the reliability of the 3-21G calculations, a single-point energy calculation was performed at a mixed basis set level, using B3LYP/6-31G(d) for all atoms except titanium, for which LANL2DZ was used^42^. This procedure was applied to the isolated Cs/GO/TiO_2_ composite, the SMX molecule, and their interacting systems. Figure 2 illustrates the optimized model structures for the Cs/GO/TiO_2_ composite and its interaction with sulfamethoxazole (SMX). Figure 2a represents the optimized Cs/GO/TiO_2_ composite, Fig. 2b shows the SMX molecule, while Fig. 2c represents the hydrated SMX model with 3 H_2_O molecules. Two possible interaction configurations were investigated. Cs/GO/TiO_2_/SMX interaction was modeled as a chemical complex, where SMX is hydrated with three H_2_O molecules. The hydrated SMX forms coordination-type bonds with the active sites of the composite. In the first configuration (Fig. 2d), SMX interacts with the composite through the amine group (–NH_2_) of chitosan. In the second configuration (Fig. 2e), the nitrogen atom of the isoxazole ring interacts with the titanium atom of TiO_2_. These two configurations were selected based on the high electron-donating ability of the amine group and the strong coordination tendency of the isoxazole nitrogen toward transition metal centers.

The calculated TDM and ΔE for the isolated and interacting systems are summarized in Table 2. As shown in Table 2, the TDM significantly increased upon SMX interaction, particularly for the configuration involving the amine group of Cs (36.831 Debye), accompanied by a marked decrease in ΔE to 0.171 eV. This implies enhanced polarity and charge redistribution, suggesting strong electronic coupling between SMX and the composite surface.

**Table 2:** Total dipole moment (TDM) and HOMO–LUMO energy gap (ΔE) for the Cs/GO/TiO_2_ composite, SMX molecule, and their interaction complexes.

Structures	TDM (Debye)	ΔE (eV)
**Cs/GO/TiO**_**2**_	25.715	0.269
**SMX**	8.326	0.931
**Cs/GO/TiO**_**2**_**/SMX via NH**_**2**_ **of Cs**	36.831	0.171
**Cs/GO/TiO**_**2**_**/SMX via TiO**_**2**_ **(N- Isoxazole)**	34.882	0.542

### Frontier molecular orbitals FMO

**Fig. 3:**
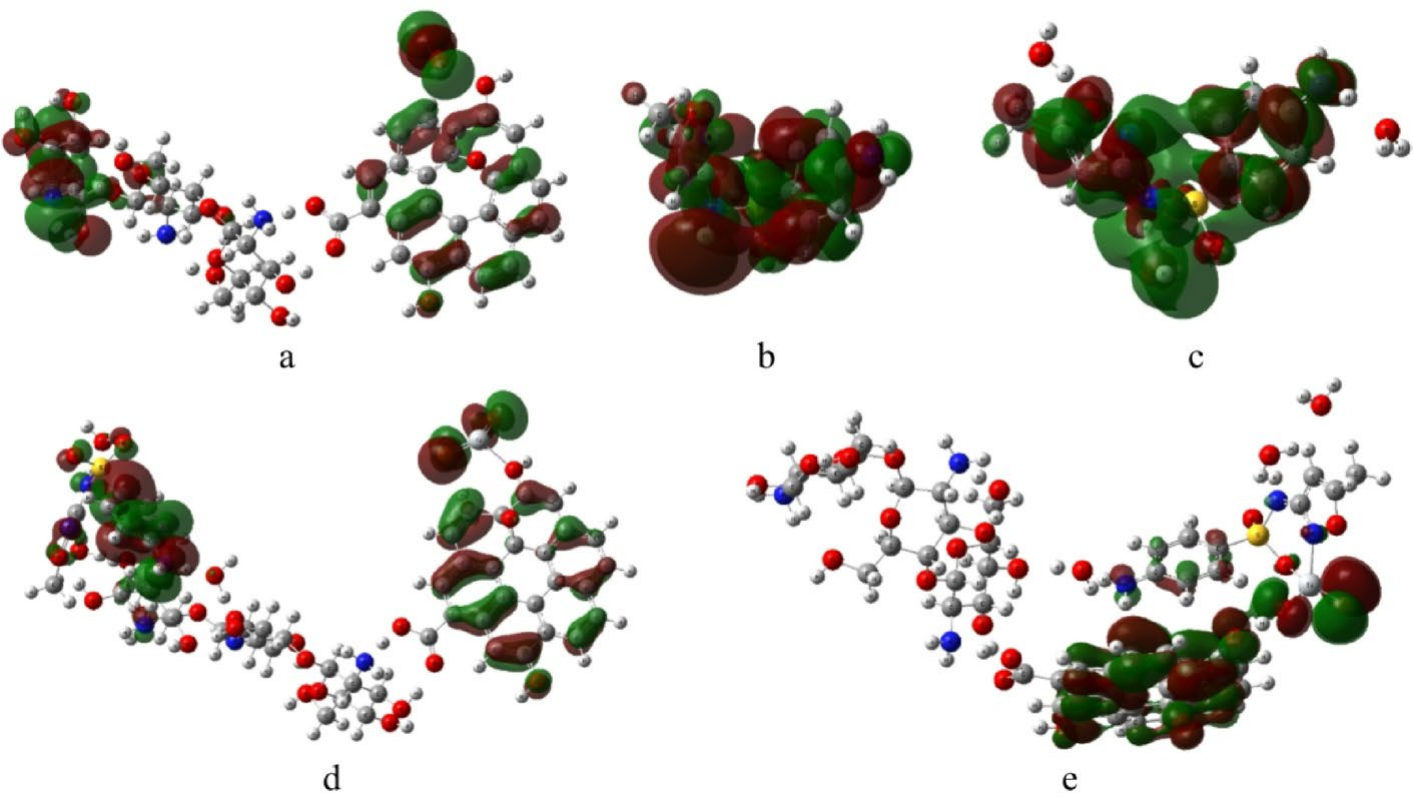
HOMO/LUMO frontier orbitals of **(a)** Cs/GO/TiO_2_ composite, **(b)** sulfamethoxazole (SMX), **(c)** Hydrated SMX, **(d)** Cs/GO/TiO_2_ interacting with SMX via the –NH_2_ group of chitosan, and **(e)** Cs/GO/TiO_2_ interacting with SMX via TiO_2_ (N–isoxazole interaction). The structures were visualized using GaussView 6.0 (Roy Dennington, Todd A. Keith, and John M. Millam, Semichem Inc., 2016; https://gaussian.com/gaussview6/).

The frontier molecular orbitals (HOMO and LUMO) were calculated at the same level of theory to provide insights into the charge transfer characteristics and electronic behavior of the studied systems^43^. The spatial distributions of the HOMO and LUMO orbitals are shown in Fig. 3, where the red and green colors represent the positive and negative phases of the molecular orbitals, respectively. For the Cs/GO/TiO_2_ composite (Fig. 3a), the HOMO is primarily localized over the chitosan region, whereas the LUMO is mainly distributed over the TiO_2_ and GO surfaces. This separation indicates a natural charge-transfer pathway from the organic matrix toward the inorganic surface. In the Cs/GO/TiO_2_/SMX complex interacting via the –NH_2_ group of chitosan (Fig. 3b), the HOMO is predominantly distributed over SMX and TiO_2_, while the LUMO remains localized on TiO_2_ and GO. Conversely, in the configuration where SMX interacts through TiO_2_ (Fig. 3c), both the HOMO and LUMO are primarily localized on TiO_2_ and GO, with slight extension of the LUMO onto the SMX molecule. These observations suggest enhanced electronic coupling and charge-transfer efficiency upon SMX adsorption, particularly in the configuration involving the amine group of chitosan, consistent with the reduced HOMO–LUMO gap obtained from energy calculations.

### Molecular electrostatic potential MESP

The molecular electrostatic potential (MESP) was calculated at the same level of theory to visualize the charge distribution and identify the reactive sites of the studied systems. The MESP maps provide a color-coded representation of electrostatic potential, where red regions correspond to electron-rich areas (negative potential), blue regions indicate electron-deficient areas (positive potential), and green areas represent nearly neutral regions^44^.

**Fig. 4:**
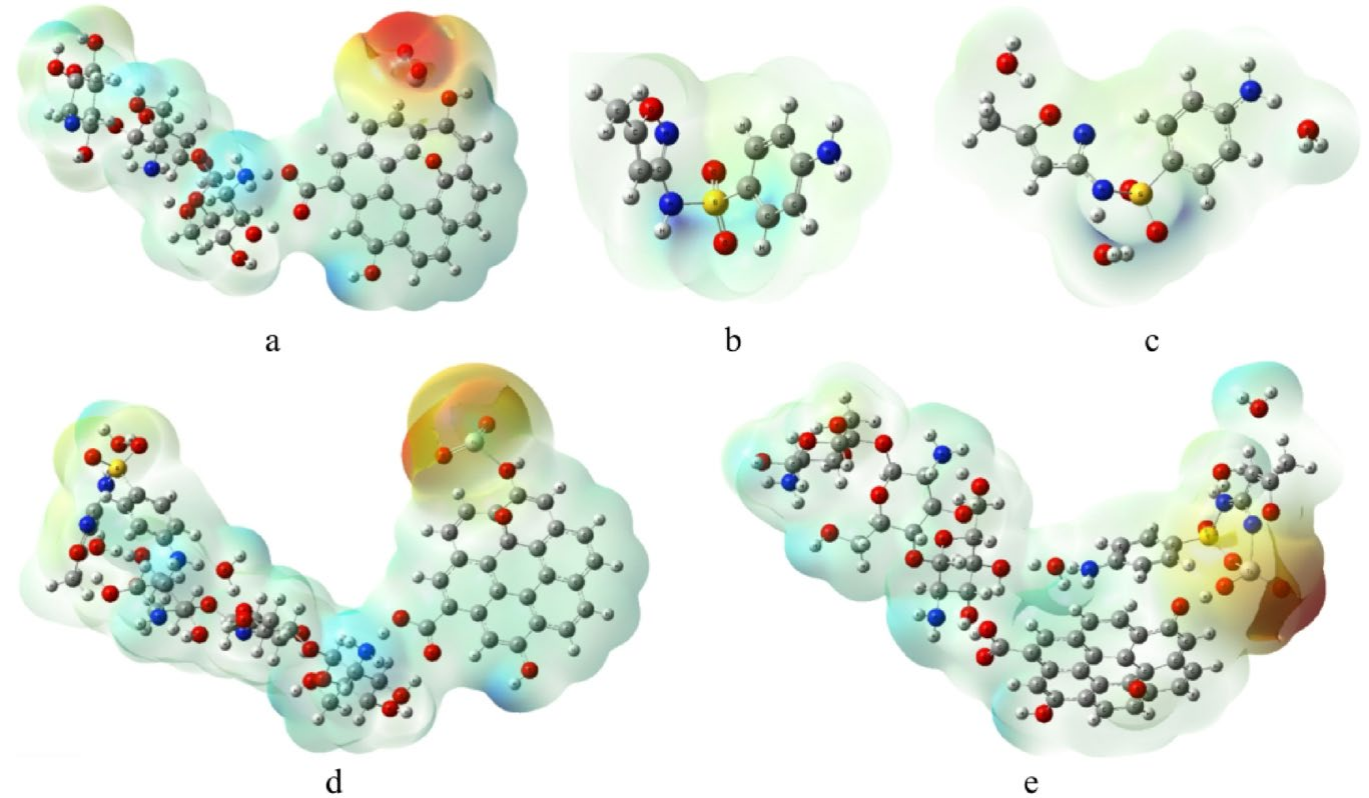
Calculated MESP of **(a)** Cs/GO/TiO_2_ composite, **(b)** sulfamethoxazole (SMX), **(c)** Hydrated SMX, **(d)** Cs/GO/TiO_2_ interacting with SMX via the –NH_2_ group of chitosan, and **(e)** Cs/GO/TiO_2_ interacting with SMX via TiO_2_ (N–N isoxazole interaction). The structures were visualized using GaussView 6.0 (Roy Dennington, Todd A. Keith, and John M. Millam, Semichem Inc., 2016; https://gaussian.com/gaussview6/).

Figure 4 presents the calculated MESP surfaces for the investigated structures. As shown in Fig. 4a, the Cs/GO/TiO_2_ composite exhibits distinct red regions around the TiO_2_ surface, indicating electron-rich areas capable of interacting with electrophilic sites, while the hydroxyl hydrogens display blue regions associated with positive potential. The SMX molecule (Fig. 4b) shows a relatively uniform potential surface with a moderately positive region localized around its nitrogen atom. After SMX adsorption, both Cs/GO/TiO_2_/SMX complexes (Fig. 4c and d) maintain similar red regions around the TiO_2_ surface, although with slightly reduced intensity. This observation suggests that the electron distribution around TiO_2_ remains largely preserved after complex formation, implying that the interaction with SMX occurs through localized charge transfer rather than significant charge redistribution across the composite surface.

### Mulliken population analysis

**Fig. 5:**
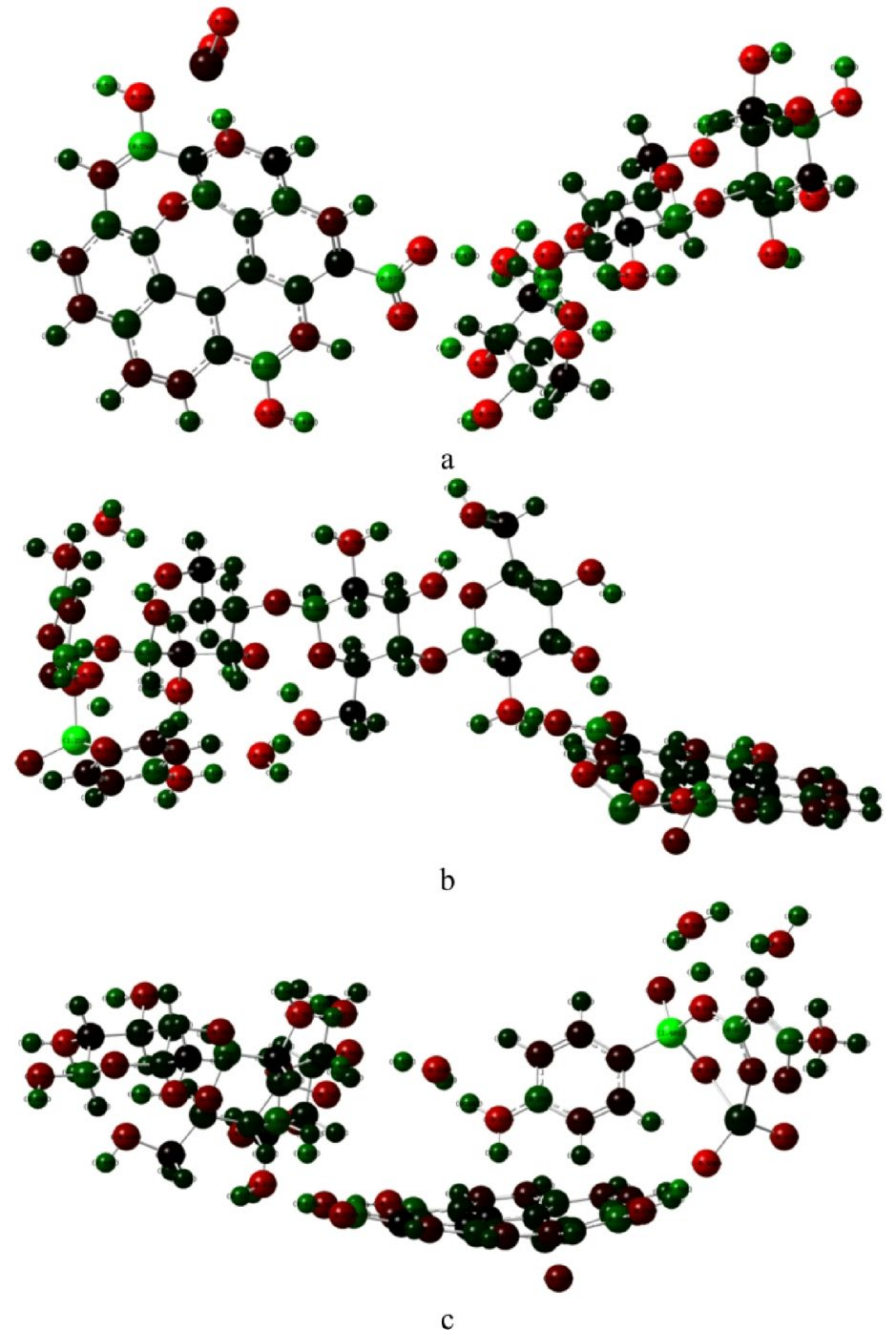
Charge distribution of **(a)** Cs/GO/TiO_2_ composite, **(b)** Cs/GO/TiO_2_ interacting with SMX via the –NH_2_ group of chitosan, and **(c)** Cs/GO/TiO_2_ interacting with SMX via TiO_2_ (N-isoxazole interaction). The structures were visualized using GaussView 6.0 (Roy Dennington, Todd A. Keith, and John M. Millam, Semichem Inc., 2016; https://gaussian.com/gaussview6/).

Mulliken population analysis was employed to clarify how the electronic structure of the Cs/GO/TiO_2_ nanocomposite responds to the adsorption of SMX through different active sites. The charge-distribution maps for the pristine composite and the two adsorption complexes provide clear evidence of charge redistribution and interaction mechanisms. Figure 5 shows Mulliken population analysis maps for Cs/GO/TiO_2_ composite and its interaction with SMX. In the pristine Cs/GO/TiO_2_ structure, the electron density is distributed heterogeneously across the composite. GO and TiO_2_ oxygen atoms exhibit high electron density, whereas Ti centers remain electron-deficient, consistent with their Lewis-acidic nature. Chitosan contains polar –NH_2_ and –OH groups, but without SMX interaction, the charge environment remains mainly balanced. This baseline polarization indicates the presence of well-defined electron-donating and electron-accepting sites that can facilitate pollutant adsorption.

Upon adsorption of SMX through the chitosan –NH_2_ group, a significant local redistribution of electron density is observed around the amine moiety. The –NH_2_ group becomes more electron-deficient, while adjacent atoms in the chitosan backbone accumulate electron density. This pattern suggests strong hydrogen bonding and dipole–dipole interactions between Cs and the SMX functional groups. The localized nature of the shifts indicates that the chitosan–SMX interaction is primarily driven by polar forces, with limited long-range electronic perturbation of the composite. A more pronounced charge redistribution occurs when SMX interacts with the TiO_2_ surface through the N-isoxazole ring. Ti centers become increasingly electron-deficient, and oxygen atoms in both TiO_2_ and the SMX ring gain electron density. This redistribution reflects a more substantial donor–acceptor interaction, in which electron density is transferred from the heteroatoms of SMX to the electron-poor Ti sites. The magnitude and spatial extent of the electronic reorganization indicate that TiO_2_ acts as a dominant adsorption center and that this interaction exhibits a higher degree of charge-transfer character than the chitosan-mediated pathway.

Overall, the Mulliken charge analysis confirms that SMX adsorption induces measurable electronic rearrangement throughout the composite, with the TiO_2_–SMX interaction being the most electronically significant. These results highlight the multi-active-site nature of the Cs/GO/TiO_2_ system, in which both the polar functionalities of chitosan and the Lewis-acidic Ti sites contribute to overall adsorption performance, albeit through different mechanisms and strengths.

### Global reactivity descriptors GRDs

Global reactivity descriptors (GRDs) provide valuable insight into the electronic behavior and chemical reactivity of molecular systems. They are derived from the energies of the frontier molecular orbitals (HOMO and LUMO) and help describe a molecule’s tendency to donate or accept electrons, its stability, and its reactivity in chemical interactions. The calculated GRDs for the studied systems are summarized in Table 3.

From Table 2, it is evident that the adsorption of sulfamethoxazole (SMX) modifies the electronic properties of Cs/GO/TiO_2_. In Table 3, the NH_2_-linked complex shows the lowest hardness (0.085 eV) and highest softness (5.848 eV⁻¹), indicating that it is the most chemically reactive and capable of efficient charge transfer. Its high electrophilicity (ω = 237.587 eV) further suggests a strong ability to accept electrons from SMX during adsorption. In contrast, the TiO_2_-bound complex exhibits greater hardness (0.271 eV) and lower softness (1.845 eV⁻¹), suggesting a more stable but less reactive configuration. The parent Cs/GO/TiO_2_ composite lies intermediate between the two. Overall, the results imply that while the TiO_2_ coordination site favors strong, localized adsorption, the NH_2_-functionalized site enhances charge-transfer reactivity, which may play a significant role in photocatalytic degradation pathways.

**Table 3:** Calculated global reactivity descriptors GRDs for the studied structures.

Structure	IP	EA	χ	µ	η	S	ω
**Cs/GO/TiO**_**2**_	6.737	6.469	6.603	-6.603	0.134	3.731	162.335
**Cs/GO/TiO**_**2**_ **/SMX via NH**_**2**_	6.452	6.281	6.367	-6.367	0.085	5.848	237.587
**Cs/GO/TiO**_**2**_ **/SMX via TiO**_**2**_	5.683	5.141	5.412	-5.412	0.271	1.845	54.001

### Total density of states TDOS

**Fig. 6:**
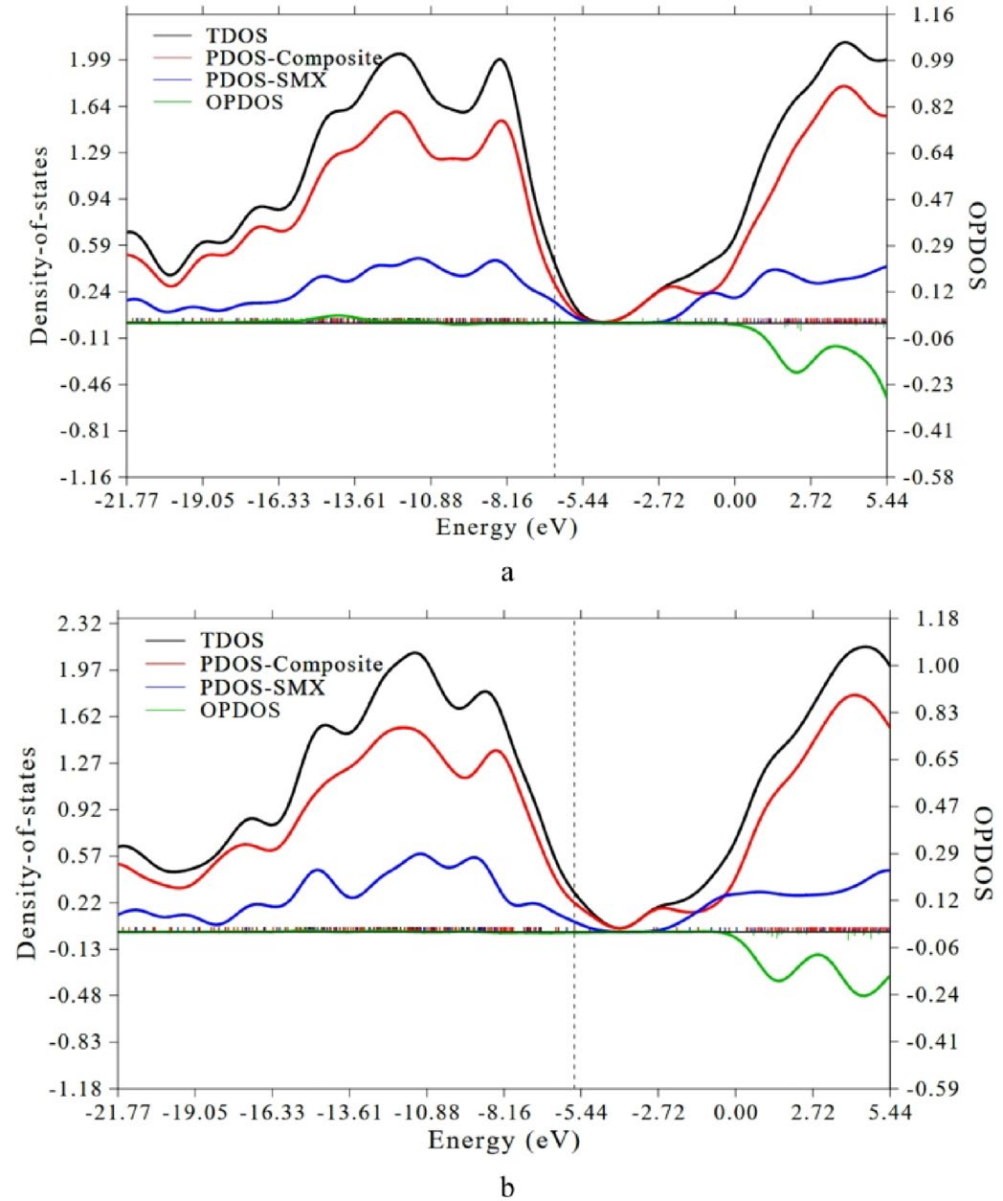
Calculated total density of states TDOS, partial density of states PDOS, and overlap density of states OPDOS plots for the Cs/GO/TiO_2_/SMX complexes, where **(a)** Cs/GO/TiO_2_ interacts with SMX via the –NH_2_ group of chitosan, and **(b)** Cs/GO/TiO_2_ interacts with SMX via TiO_2_ (N–isoxazole interaction). The TDOS spectra were generated using the Multiwfn program (Lu & Chen, J. Comput. Chem. 33, 580–592, 2012) http://sobereva.com/multiwfn/index.html.

The total density of states (TDOS), partial density of states (PDOS), and overlap population density of states (OPDOS) provide valuable insights into the electronic structure and nature of interaction between the composite and the adsorbed SMX molecules. The spectra were generated using Multiwfn software^38^. The TDOS describes the overall distribution of electronic states over the energy levels, while the PDOS identifies the contributions of individual fragments (composite and SMX) to these states. The OPDOS, on the other hand, reflects the degree of orbital overlap between fragments, indicating bonding (positive values) or antibonding (negative values) interactions. The Fermi level (set at 0 eV) serves as the reference energy separating filled and unfilled molecular orbitals. Figure 6 displays the TDOS, PDOS, and OPDOS plots for both adsorption configurations of hydrated SMX on Cs/GO/TiO_2_.

For the Cs/GO/TiO_2_–SMX complex through the –NH_2_ group of chitosan (Fig. 6a), the TDOS profile shows a noticeable overlap between the PDOS of the composite (red curve) and that of SMX (blue curve) near the Fermi level, implying significant orbital hybridization and charge redistribution upon complex formation. The OPDOS (green curve) exhibits weakly negative oscillations near the Fermi level, reflecting a mixture of bonding and minor antibonding interactions. This pattern supports the formation of a partially covalent or coordinate bond between the –NH_2_ site of chitosan and the composite surface, stabilized by weak noncovalent interactions. In contrast, for the Cs/GO/TiO_2_/SMX complex via TiO_2_ (N–isoxazole interaction) (Fig. 6b), the PDOS contributions show a similar but slightly weaker overlap in the frontier region, indicating that the electronic coupling between SMX and TiO_2_ is somewhat less intense. The TDOS peaks in both valence and conduction regions are smoother and less pronounced near the Fermi level, suggesting limited orbital delocalization compared to the –NH_2_ complex.

Overall, the DOS analysis corroborates that both adsorption configurations involve electronic hybridization between SMX and the composite. However, the –NH_2_-linked complex exhibits stronger orbital mixing, reduced band gap, and greater charge transfer character features that reinforce its higher adsorption stability and better interaction strength.

### Removal energy E_ads_

The adsorption (removal) energies $\left({E}_{\mathrm{ads}}\right)$ were calculated using the relation:

$${E}_{\mathrm{ads}}={E}_{\mathrm{complex}}-\left({E}_{\mathrm{composite}}+{E}_{\mathrm{SMX}}\right)$$

where ${E}_{\mathrm{complex}}$, ${E}_{\mathrm{composite}}$, and ${E}_{\mathrm{SMX}}$ are the total electronic energies of the adsorbed system, the isolated composite, and the free SMX molecule, respectively. All calculations were performed at the B3LYP/3-21G level and recorded in Table 4.

The obtained adsorption energies were − 2.31 eV (− 222.95 kJ·mol⁻¹) for the Cs/GO/TiO_2_/SMX complex via the –NH_2_ group of chitosan, and − 3.69 eV (− 356.09 kJ·mol⁻¹) for the configuration involving coordination between the Ti center and the isoxazole nitrogen of SMX. The negative values confirm that both adsorption processes are exothermic and energetically favorable.

The large magnitudes of the adsorption energies, exceeding 2 eV in both cases, clearly indicate that the interactions correspond to strong binding rather than weak physical adsorption. The stronger adsorption energy observed for the Ti–N configuration suggests a more stable complex formation through coordination bonding between the Ti site and the nitrogen atom of SMX. In contrast, the –NH_2_-linked configuration, while slightly less exothermic, still indicates strong chemical interaction and efficient immobilization of SMX onto the composite surface.

These exothermic adsorption energies are consistent with DFT calculations for the removal of sulfamethazine (a structurally similar sulfonamide) on chitosan-functionalized 3D graphene oxide, where physisorption pathway was identified as feasible, driven by van der Waals forces, H-bonding, and π-π interactions^10^.

These results collectively support the formation of stable adsorption complexes between SMX and the Cs/GO/TiO_2_ composite, dominated by chemical bonding and charge transfer rather than simple van der Waals interactions.

**Table 4:** Calculated removal energy E_ad_ as eV for SMX adsorbed onto Cs/GO/TiO_2_.

Structure	Adsorption energy (eV)
**Cs/GO/TiO**_**2**_**/SMX via NH**_**2**_ **of Cs**	-2.311
**Cs/GO/TiO**_**2**_**/SMX via TiO**_**2**_ **(N- Isoxazole)**	-3.691

### Quantum theory of atoms in molecules QTAIM

The Quantum Theory of Atoms in Molecules (QTAIM) provides a powerful framework for analyzing chemical bonding and intermolecular interactions, including non-covalent interactions. QTAIM is based on the topological analysis of the electron density, ρ(r), which represents the probability of finding an electron at a given point in space^45,46^. By mapping ρ(r) and examining its critical points, especially bond critical points (BCPs), one can identify the nature and strength of interactions within and between molecules. The Laplacian of electron density (∇²ρ) differentiates regions of charge concentration (∇²ρ < 0), associated with covalent bonding, from regions of charge depletion (∇²ρ > 0), typical of ionic or van der Waals interactions. Additionally, the total energy density, H(r), which combines potential and kinetic energy densities, provides insight into bond character: negative H(r) values indicate shared-shell (covalent) interactions, whereas positive values correspond to closed-shell (electrostatic) interactions^45,46^.

**Fig. 7:**
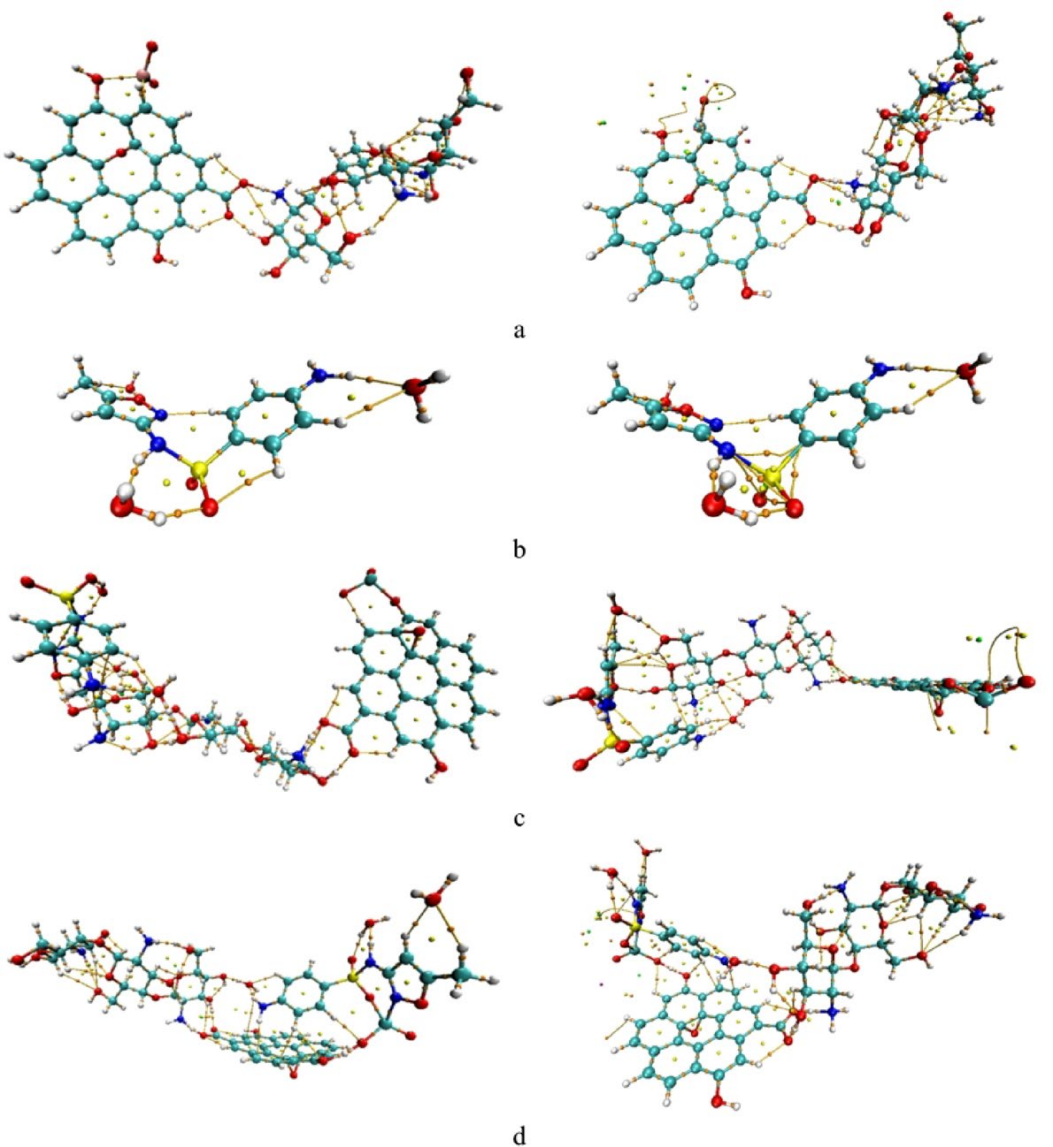
Calculated QTAIM topology before and after dispersion correction respectively, of **(a)** Cs/GO/TiO_2_ composite, **(b)** Hydrated sulfamethoxazole (SMX), **(c)** Cs/GO/TiO_2_ interacting with SMX via the –NH_2_ group of chitosan, and **(d)** Cs/GO/TiO_2_ interacting with SMX via TiO_2_ (N–isoxazole interaction). Visualization of QTAIM topology was performed using VMD 1.9.3 (William Humphrey, Andrew Dalke, and Klaus Schulten, University of Illinois at Urbana–Champaign, 2016; http://www.ks.uiuc.edu/Research/vmd/.

Figure 7 illustrates the QTAIM topology for the nanocomposite and its interaction with SMX. QTAIM analysis of the Cs/GO/TiO_2_/SMX systems reveals a spectrum of interaction types. In hydrated SMX, several intramolecular and hydration O–H···O hydrogen bonds are present; some (ρ ≈ 0.06–0.07 a.u., H(r) < 0) display strong, partially covalent character, while weaker hydrogen bonds (ρ ≈ 0.01–0.03 a.u., H(r) > 0) are predominantly electrostatic. In the pristine composite, Ti–O and Ti–N contacts (ρ ≈ 0.07–0.08 a.u., H(r) < 0) exhibit partial covalent or coordinate bonding characteristic of metal–ligand interaction. For the two modeled adsorption complexes, QTAIM identifies multiple interfacial hydrogen bonds and a direct H···N contact at the NH_2_ site with moderate electron density (ρ ≈ 0.02–0.06 a.u.) and negative H(r), consistent with strong hydrogen bonding with partial covalent character. Overall, QTAIM supports the conclusion that adsorption proceeds via formation of a chemically bound complex with mixed charge-transfer and coordination bonding character; these findings agree with the adsorption energies, DOS, and HOMO/LUMO analyses.

To further refine the topological description of weak interactions, QTAIM was re-evaluated using D3-corrected electron densities. Dispersion-corrected densities reveal additional weak BCPs around the TiO_2_ region and at the composite–SMX interface, reflecting enhanced long-range stabilization not captured in the uncorrected topology.

### Non-covalent interactions NCI and reduced density gradient RDG

While the QTAIM results confirmed that adsorption between SMX and the Cs/GO/TiO_2_ surface proceeds mainly through covalent or coordination-type bonding, Non-Covalent Interaction (NCI) and Reduced Density Gradient (RDG) analyses were conducted to visualize the complementary weak interactions that further stabilize these complexes. NCI and RDG methods identify regions of non-covalent attraction or repulsion based on the reduced density gradient *s(r)* and the sign of the second Hessian eigenvalue multiplied by the electron density [sign (λ_2_) ρ]. Blue regions correspond to attractive interactions (such as hydrogen bonding), green regions indicate weak van der Waals forces, and red regions represent steric repulsion.

**Fig. 8:**
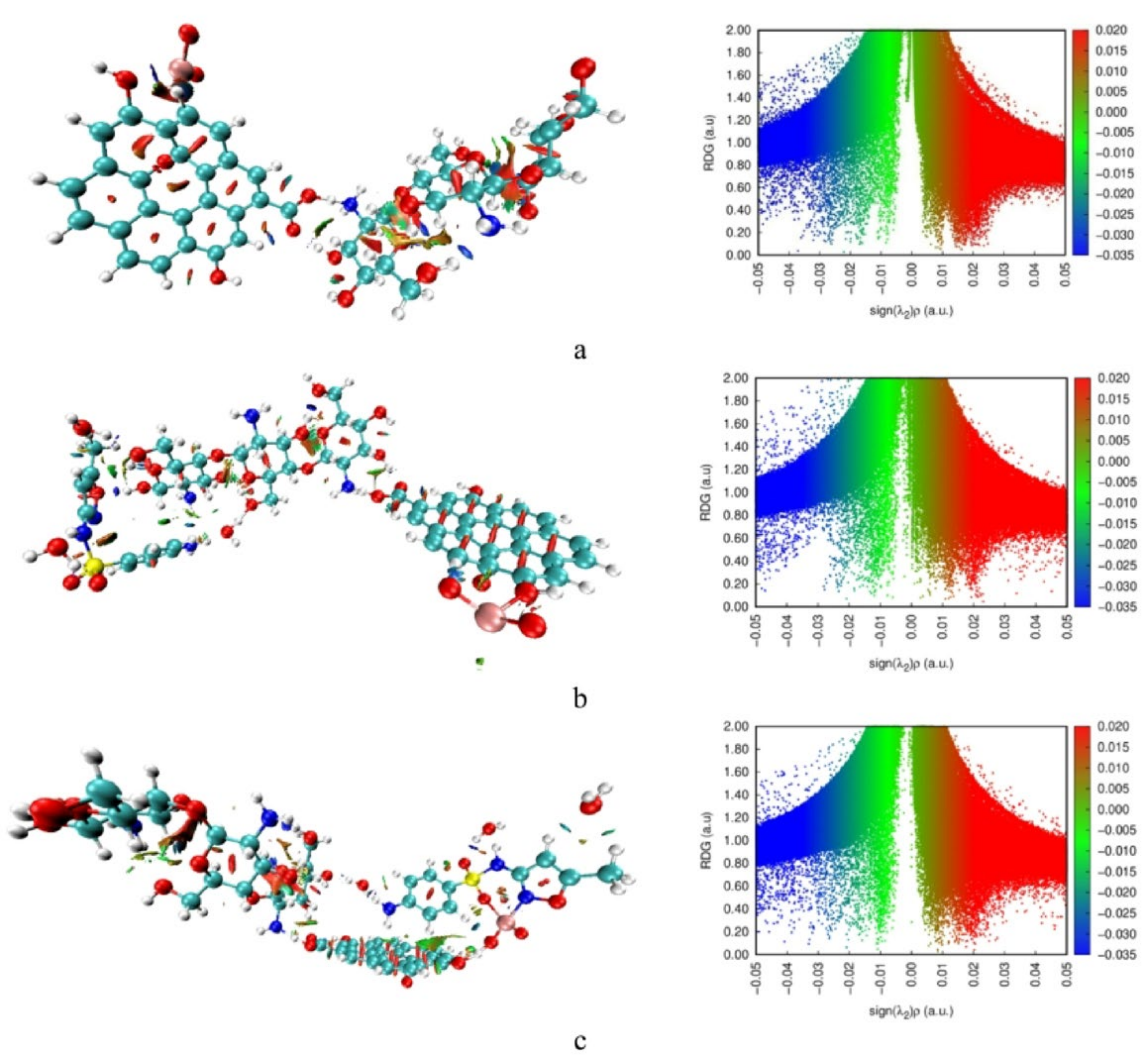
Calculated NCI and RDG plots of **(a)** Cs/GO/TiO_2_ composite, **(b)** Cs/GO/TiO_2_ interacting with SMX via the –NH_2_ group of chitosan, and **(c)** Cs/GO/TiO_2_ interacting with SMX via TiO_2_ (N–isoxazole interaction). Visualization was generated using VMD 1.9.3 (William Humphrey, Andrew Dalke, and Klaus Schulten, University of Illinois at Urbana–Champaign, 2016; http://www.ks.uiuc.edu/Research/vmd/.

As illustrated in Fig. 8, the RDG plots of all systems display distinct green spikes, confirming the presence of van der Waals interactions between SMX and the Cs/GO/TiO_2_ composite. The system where SMX interacts via the –NH_2_ group of chitosan exhibits slightly stronger blue spikes compared to the pristine composite and the TiO_2_-linked configuration, suggesting the existence of additional hydrogen-bonding interactions that enhance complex stability. The NCI isosurfaces further support these observations, showing blue and green regions distributed along the interface, corresponding to hydrogen bonding and dispersive forces.

**Fig. 9:**
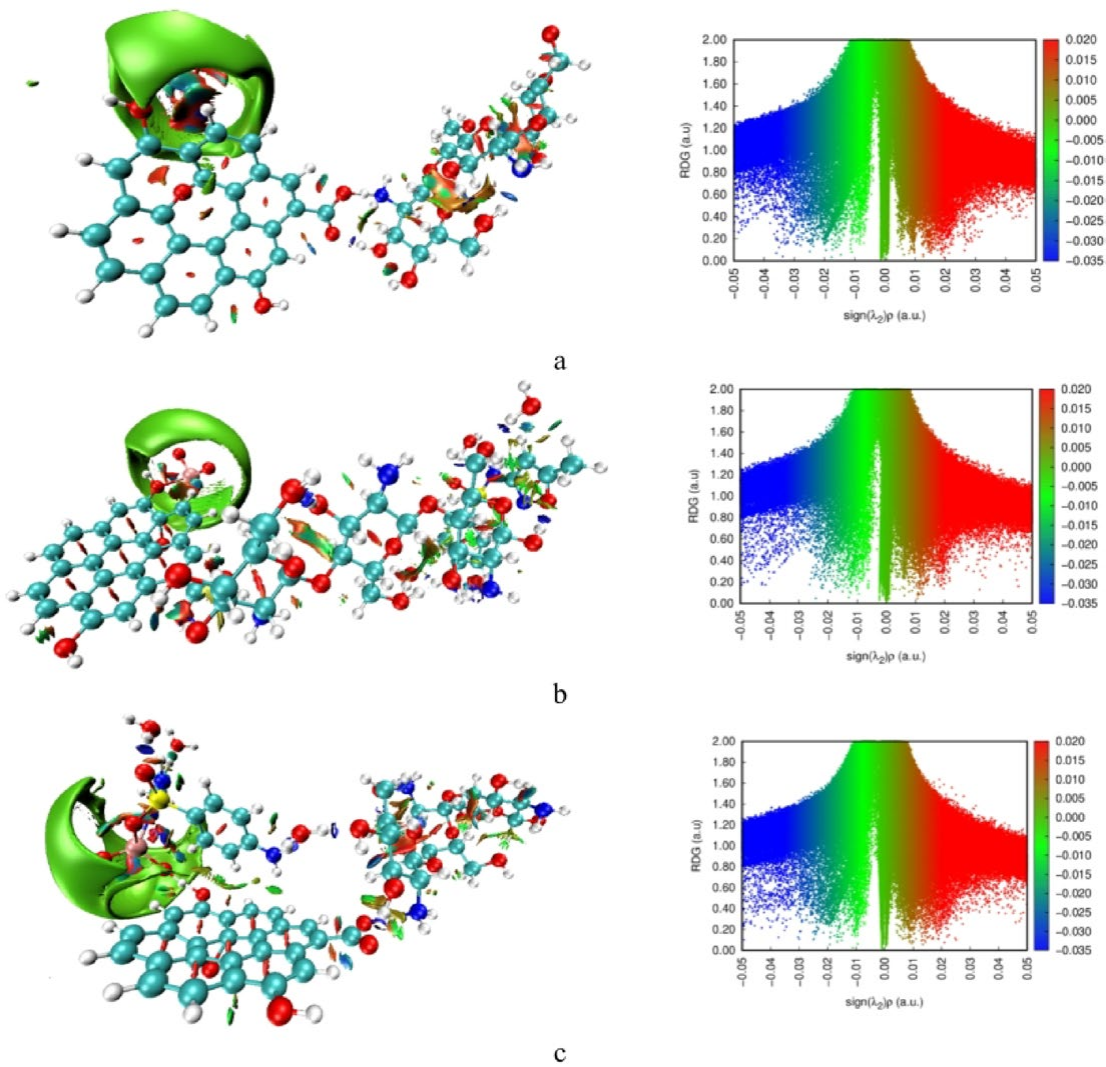
Dispersion-corrected NCI isosurfaces and RDG plots obtained using D3-corrected electron densities of **(a)** Cs/GO/TiO_2_ composite, **(b)** Cs/GO/TiO_2_ interacting with SMX via the –NH_2_ group of chitosan, and **(c)** Cs/GO/TiO_2_ interacting with SMX via TiO_2_ (N–isoxazole interaction). Visualization was generated using VMD 1.9.3 (William Humphrey, Andrew Dalke, and Klaus Schulten, University of Illinois at Urbana–Champaign, 2016; http://www.ks.uiuc.edu/Research/vmd/.

To assess the influence of dispersion on these weak-interaction features, NCI and RDG analyses were performed both without and with Grimme-type D3 dispersion. The dispersion-corrected results in Fig. 9 display a clearer and more extensive visualization of van der Waals regions, including the appearance of a pronounced green isosurface around the TiO_2_ domain that persists even after SMX adsorption. In the RDG plots, the D3-corrected density introduces additional green spikes, indicating enhanced dispersion contributions that were not captured in the uncorrected calculations. These new features reflect the expected strengthening of long-range correlation effects around the Ti centers and at the composite/SMX interface.

The enlarged green NCI isosurfaces and additional RDG spikes observed after dispersion correction corroborate the extra weak critical points identified in the QTAIM analysis, confirming stronger van der Waals contributions in these regions.

Overall, the NCI and RDG analyses, particularly after incorporating dispersion, provide a more refined depiction of the weak interactions governing SMX adsorption. Together, these density-based descriptors complement the QTAIM findings and confirm that, beyond the primary covalent or coordination-type bonding, dispersion and hydrogen-bonding interactions play a significant role in stabilizing the Cs/GO/TiO_2_/SMX complexes^47^.

### Limitations of the study

The present DFT investigation is subject to several limitations inherent to the adopted computational approach. Finite cluster models were used to represent the Cs/GO/TiO₂ composite, which do not fully capture long-range periodic surface effects. Explicit solvent dynamics were not included, and adsorption energies were evaluated in the absence of basis set superposition error (BSSE) correction; therefore, absolute adsorption energies may be quantitatively overestimated. In addition, the calculations rely on the B3LYP functional combined with a modest basis set, which may influence the numerical accuracy of the energetic values. Dispersion interactions were incorporated only at the density level for QTAIM and NCI/RDG analyses, whereas geometry optimizations and adsorption energy calculations were performed without explicit dispersion correction. Furthermore, Mulliken population analysis was employed only for qualitative charge redistribution analysis and is known to be sensitive to basis set selection. Consequently, the energetic conclusions are based on non-dispersion-corrected geometries, while dispersion effects are discussed qualitatively. Despite these limitations, the relative stability trends and comparative interaction mechanisms between adsorption configurations remain reliable.

## Conclusion

This DFT investigation provides qualitative insight into the adsorption mechanism of hydrated sulfamethoxazole (SMX) on Cs/GO/TiO_2_ composites through two primary configurations: the chitosan –NH_2_ group and TiO_2_ coordination. Both exhibited exothermic adsorption, with energies of − 2.31 and − 3.69 eV, respectively. Electronic analyses (TDM, ΔE, MESP, FMO, GRDs, DOS) indicated significant charge transfer and polarization, particularly in the –NH_2_-linked complex, which showed a reduced band gap (0.171 eV), high softness (5.848 eV⁻¹), and strong electrophilicity (ω = 237.59 eV). Mulliken population analysis further revealed distinct pathways of charge redistribution upon adsorption, confirming that the TiO_2_ surface undergoes the most pronounced electron-density reorganization during SMX binding. QTAIM analysis confirmed a mixture of covalent and electrostatic bonding, while NCI/RDG visualization revealed complementary stabilization via hydrogen bonding and van der Waals interactions. The inclusion of dispersion-corrected density analyses further refined the description of these weak interactions, providing a more accurate picture of the interfacial stabilization. While the absolute adsorption energies are model-dependent, the observed relative stability trends and interaction characteristics highlights how the synergistic interaction among Cs, GO, and TiO_2_ enhances adsorption stability and electronic reactivity toward SMX. This atomic-scale insight advances the rational design of multifunctional Cs/GO/TiO_2_ composites for removal of antibiotic contaminants from aqueous environments.

## Data Availability

The data that support the findings of this study are available from the corresponding author upon reasonable request.
